# A method for *in vivo* identification of bacterial small RNA-binding proteins

**DOI:** 10.1002/mbo3.220

**Published:** 2014-10-29

**Authors:** Jonathan Osborne, Louise Djapgne, Bao Quoc Tran, Young Ah Goo, Amanda G Oglesby-Sherrouse

**Affiliations:** 1 Department of Pharmaceutical Sciences, School of Pharmacy, University of MarylandBaltimore, Maryland; 2 Department of Microbiology and Immunology, School of Medicine, University of MarylandBaltimore, Maryland

**Keywords:** Heme regulation, iron regulation, PrrF, PrrH, *Pseudomonas aeruginosa*, small RNAs

## Abstract

Small bacterial regulatory RNAs (sRNAs) have gained immense appreciation over the last decade for their roles in mediating posttranscriptional gene regulation of numerous physiological processes. Several proteins contribute to sRNA stability and regulation, most notably the Hfq RNA-binding protein. However, not all sRNAs rely on Hfq for their stability. It is therefore likely that other proteins contribute to the stability and function of certain bacterial sRNAs. Here, we describe a methodology for identifying *in vivo*-binding proteins of sRNAs, developed using the iron-responsive PrrF and PrrH sRNAs of *Pseudomonas aeruginosa*. RNA was isolated from iron-depleted cultures, which were irradiated to cross-link nucleoprotein complexes. Subsequently, PrrF- and PrrH-protein complexes were enriched using cDNA “bait”, and enriched RNA-protein complexes were analyzed by tandem mass spectrometry to identify PrrF and PrrH associated proteins. This method identified Hfq as a potential PrrF- and PrrH-binding protein. Interestingly, Hfq was identified more often in samples probed with the PrrF cDNA “bait” as compared to the PrrH cDNA “bait”, suggesting Hfq has a stronger binding affinity for the PrrF sRNAs *in vivo*. Hfq binding to the PrrF and PrrH sRNAs was validated by electrophoretic mobility shift assays with purified Hfq protein from *P. aeruginosa*. As such, this study demonstrates that *in vivo* cross-linking coupled with sequence-specific affinity chromatography and tandem mass spectrometry (SSAC-MS/MS) is an effective methodology for unbiased identification of bacterial sRNA-binding proteins.

## Introduction

Small noncoding RNAs (sRNAs) have emerged over the past decade as central regulators of gene expression involved in a wide variety of bacterial processes, including carbon utilization, iron homeostasis, quorum sensing, and virulence (Bobrovskyy and Vanderpool 2013; Caldelari et al. 2013; Oglesby-Sherrouse and Murphy 2013; Romeo et al. 2013; Shao et al. 2013; Gruber and Sperandio 2014). In general, the bacterial sRNAs that have been described to date can be divided into two families – sRNAs that pair with complementary messenger RNAs (mRNAs), and sRNAs that interact with posttranscriptional regulatory proteins. sRNAs that pair with mRNAs can be encoded antisense to their target mRNAs (*cis*-antisense sRNAs) or at distal genetic loci (*trans*-encoded sRNAs). Pairing with members of this class of sRNAs leads to either stabilization and/or increased translation efficiency, or destabilization and/or decreased translation, of the targeted mRNA (Gottesman et al. 2006; Frohlich and Vogel 2009; Soper et al. 2010; Gottesman and Storz 2011; Bobrovskyy and Vanderpool 2013; Caldelari et al. 2013). In contrast to *trans*-acting sRNAs, the Csr/Rsm family of sRNAs function by binding to and sequestering posttranscriptional regulatory proteins from their target mRNAs (Lucchetti-Miganeh et al. 2008; Heroven et al. 2012; Romeo et al. 2013). In spite of this generalized division of bacterial sRNAs, mounting evidence suggests sRNAs can modulate gene expression by a myriad of molecular mechanisms.

One factor required for the function and stability of many bacterial sRNAs is the host factor for bacteriophage Q_*β*_, or Hfq (Aiba 2007; Vogel and Luisi 2011). Hfq was originally identified in 1965 in *Escherichia coli* as a factor required for replication of RNA phages (Haruna and Spiegelman 1965), but has since been shown to mediate regulation of transcriptional termination, translation, and RNA stability (Vogel and Luisi 2011). While Hfq is known to mediate the stability and function of many bacterial sRNAs, notable exceptions to this paradigm exist. For example, the iron-responsive RyhB sRNAs of Yersinia species, which are duplicated in the bacteria of this genus, have different stability requirements for Hfq (Deng et al. 2012). Moreover, the Gram-positive bacterium *Bacillus subtilis* encodes for an iron-responsive sRNA, named FsrA, whose stability and function are independent of the *B. subtilis* hfq homolog (Gaballa et al. 2008). Instead, FsrA activity appears to be modulated by three small basic proteins, FbpA, B, and C (Smaldone et al. 2012a, 2012b). Among many others, these studies demonstrate the complexity of protein requirements guiding sRNA stability and function in different bacterial species, and highlight the need for further study into the proteins that contribute to sRNA regulation.

Iron is an essential metallo-nutrient for most organisms, and bacteria have evolved several strategies to mediate its uptake and utilization (Andrews et al. 2003). Iron can also be toxic due to its ability to catalyze the formation of reactive oxygen species via Fenton chemistry. Thus, bacterial iron and heme uptake systems are tightly regulated in response to intracellular iron concentrations (Andrews et al. 2003). In many bacteria, this regulation is mediated by the iron-binding ferric uptake repressor (Fur) (Hantke 2001), which blocks expression of genes coding for iron acquisition systems in iron-replete environments. The Fur protein has also been shown to mediate positive regulation of gene expression in multiple bacterial species. In some cases, this regulation occurs by direct interaction of the Fur protein with the promoters of target genes (Faulkner et al. 2012). In 2002, Masse and Gottesman (2002) described the first example of a Fur-regulated small regulatory RNA (sRNA), named RyhB, which mediates a large proportion of positive regulation by Fur in *E. coli*. RyhB regulation was subsequently shown to contribute to *E. coli* iron homeostasis by blocking the expression of nonessential iron-containing proteins, thereby sparing limiting iron stores for more essential functions (Masse et al. 2005; Jacques et al. 2006). Iron-regulated sRNAs are now known to be widespread in bacteria, having been identified in both Gram-positive and Gram-negative bacterial species (Salvail and Masse 2012; Oglesby-Sherrouse and Murphy 2013).

*Pseudomonas aeruginosa*, a ubiquitous Gram-negative opportunistic pathogen, requires iron for infection (Cox 1982; Meyer et al. 1996; Takase et al. 2000a, 2000b; Xiong et al. 2000; Nadal Jimenez et al. 2010) and has evolved a complex hierarchical regulatory system to mediate iron homeostasis (Poole and McKay 2003). As part of this system, the *P. aeruginosa* Fur protein blocks the expression of two nearly identical regulatory sRNAs, named PrrF1 and PrrF2 for *P*seudomonas *R*NA *R*esponsive to Iron (*F*e) (Wilderman et al. 2004). The PrrF sRNAs are analogs of the RyhB sRNA in *E. coli*, and as such regulate the expression of a number genes encoding iron-containing proteins, many of which are involved in metabolism (Wilderman et al. 2004; Oglesby et al. 2008). While most Pseudomonads encode for two PrrF sRNAs, only *P. aeruginosa* encodes these sRNAs in tandem, allowing for the expression of a longer, heme-regulated sRNA named PrrH (Oglesby-Sherrouse and Vasil 2010). PrrH transcription is initiated at the *prrF1* Fur-regulated promoter, reads through the *prrF1* Rho-independent terminator and the *prrF1-prrF2* intergenic region (IGR), and terminates at the *prrF2* Rho-independent terminator. Due to its unique sequence, PrrH is predicted to interact with a distinct set of mRNAs, allowing for unique heme regulation properties in *P. aeruginosa*. To date, it remains unknown if either of the PrrF or PrrH sRNAs interact with Hfq, whether or not Hfq plays a role in their expression, stability, and function, or if additional proteins contribute to stability of and regulation by this unique group of iron-responsive sRNAs.

The goal of the current study was to identify proteins that interact with the bacterial sRNAs *in vivo*. Previous studies have expressed aptamer-tagged sRNAs to purify sRNA-protein complexes to achieve this goal (Said et al. 2009). However, this procedure requires genetic manipulations of the sRNA-encoding DNA, which could potentially affect interactions with proteins due to altered expression and/or structure. Here, we have adapted a previously developed sequence specific affinity chromatography strategy used to identify RNA-binding proteins in eukaryotic organisms (Blencowe et al. 1989; Lingner and Cech 1996) to identify proteins that interact with the natively expressed PrrF and PrrH sRNAs. Combined with *in vivo* cross-linking and tandem mass spectrometry, this method identified Hfq as a potential PrrF- and PrrH-interacting protein, which we verified by in vitro analysis using electrophoretic mobility shift assays (EMSA's) of the PrrF and PrrH sRNAs with purified Hfq. As such, this approach provides a means for unbiased identification of proteins that interact with bacterial sRNAs *in vivo*, particularly in the case of sRNAs that are not dependent upon Hfq for stability or function.

## Materials and Methods

### Bacterial strains and growth conditions

Bacterial strains used in this work are listed in Table 1. *Escherichia coli* strains were routinely grown in L broth or on L agar plates, and *P. aeruginosa* strains were maintained in brain-heart infusion (BHI) broth or on BHI agar plates. For qPCR, northern blots, and sequence-specific affinity chromatography and tandem mass spectrometry (SSAC-MS/MS) pull-down studies, strains were diluted from overnights of LB into M9 minimal media purchased from Teknova containing 2% glucose and grown for 4 h to deplete intracellular iron stores, then subcultured into fresh M9 for an additional 8 h at 37°C (Nguyen et al. 2014). Ferric chloride (FeCl_3_) was added to a final concentration of 100 *μ*mol/L where indicated. Ampicillin (100 *μ*g/mL) and chloramphenicol (12.5 *μ*g/mL) were used for growth of *E. coli* carrying the pTYB21-hfq plasmid.

**Table 1:** Bacterial strains used in this study

Strain	Description	Source or reference
PAO1	Wild-type *Pseudomonas aeruginosa* strain	Holloway (1955)
ΔprrF1,2	PAO1 strain with a deletion in the ΔprrF1,2 locus	Wilderman et al. (2004)
Rosetta™ 2 (DE3)	Derivative of BL21 (DE3) *Escherichia coli* strain designed for enhanced expression of nonnative proteins	Novagen

### Northern blots

**Figure 1:**
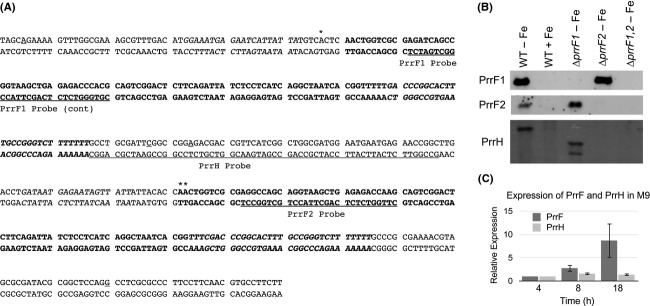
Development of PrrF and PrrH probes for sequence-specific affinity chromatography and tandem mass spectrometry (SSAC-MS/MS). (A) Sequence of the *prrF* locus, with the PrrF1, PrrF2 and PrrH probe locations underlined. Asterisks indicate the transcriptional start site of PrrF1 and PrrH (*) and PrrF2 (**). The PrrF1 and PrrF2 transcribed sequences are in bold, and the PrrF1 and PrrF2 Rho-independent terminators are italicized. (B) Northern blots of PAO1 and the indicated *prrF* mutants grown in M9 minimal media for 18 h, with or without supplementation of 100 *μ*mol/L FeCl_3_ as indicated, using the PrrF1, PrrF2, and PrrH probes shown in (A). (C) Expression of the PrrF and PrrH sRNAs was determined by qPCR after 4, 8, and 18 h of growth in M9 minimal media with no iron supplementation. Relative expression of each RNA was determined using a standard curve, and values for each time point were normalized to the 4 h time point. Error bars indicate the standard deviation of three independent experiments.

Northern analysis of the PrrF and PrrH sRNAs was performed as previously described with some modifications (Oglesby-Sherrouse and Vasil 2010). Briefly, 10–20 *μ*g of total RNA isolated on RNeasy Mini Columns was run on a 6% polyacrylamide denaturing (7 mol/L urea) gel then transferred to a BrightStar membrane (Life Technologies, Grand Island, NY, USA) using a semi-dry transfer apparatus. Biotinylated oligonucleotides that were complementary to the regions of PrrF1, PrrF2, or PrrH as shown in Figure 1A were purchased from Integrated DNA Technologies (IDT) and hybridized to blots overnight at 42°C. The membrane was washed using the Northern Max Low Stringency and High Stringency wash solutions according to the manufacturer's instructions. Detection of the biotinylated probes was carried out using the BrightStar BioDetect nonisotopic detection kit (Life Technologies).

### Real-time PCR

Real-time PCR of the PrrF and PrrH sRNAs was carried out as described previously (Nguyen et al. 2014). Briefly, RNA was extracted using the Qiagen Venlo, Limburg RNeasy Mini Kit according to manufacturer's directions. 50 ng/*μ*L of RNA was used to generate cDNA with the ImPromII cDNA synthesis kit (Promega Madison, Wisconsin, USA), and cDNA was analyzed using the StepOnePlus instrument (Life Technologies) and Taqman reagents (Life Technologies). Standard curves were produced for each primer probe set by analyzing cDNA generated from serial dilutions of RNA and used to determine relative amounts of the corresponding RNAs in each sample. Relative RNA levels in each sample were then normalized to the oprF mRNA.

### Enrichment of PrrF- and PrrH-protein complexes from irradiated cultures

Six milliliter of PAO1 iron-depleted cultures were mixed with 1.5 mL RNA-Later® (Qiagen) to stabilize and preserve RNA transcripts. The mixture was then poured into a 150 × 15 mm petri dish and placed into a VWR (Radnor, PA, USA) UV-cross-linker ˜ 4 inches away for the UV source. Cultures were then UV irradiated with a wavelength of 254 nm for 3 min. RNA was extracted from 1.25 mL of irradiated culture using Qiagen RNeasy Mini Columns according to the manufacturer's instructions. Purified RNA was combined with 1 *μ*g of 5′ biotinylated PrrF or PrrH cDNA-bait, purchased from IDT. The RNA-bait mixture was brought up to 100 *μ*L of RNase-free water and incubated at 70°C for 15 min, followed by a 37°C incubation for 15 min with gentle shaking. The RNA-bait mixture was combined with 1 mg of M-270 Dynabeads® (Life Technologies) in 100 *μ*L of binding and washing buffer (10 mmol/L Tris-HCl pH 7.5, 1 mmol/L EDTA (ethylenediaminetetraacetic acid), 2 mol/L NaCl), and the RNA-bait Dynabead® mixture was incubated at 37°C for 45 min with gentle shaking. After incubation, the beads were separated from solution using a magnetic stand, and the supernatant was removed. The beads were then resuspended in 100 *μ*L of RNase-free water and incubated at 65°C for 10 min to disrupt the streptavidin-biotin linkage. The beads were separated from the resulting solution using a magnetic stand, and the supernatant was collected for tryptic digestion and mass spectrometric analysis.

### Proteolytic digestion and desalting for RNA-protein samples

The samples were brought up to a final volume of 300 *μ*L in 6 mol/L urea and 50 mmol/L ammonium bicarbonate, then combined with 20 *μ*L of 1.5 mmol/L Tris pH 8.8 and 7.5 *μ*L of 200 mmol/L TCEP (tris(2-carboxyethyl)phosphine) and allowed to incubate for 1 h at 37°C, followed by addition of 60 *μ*L of 200 mmol/L iodoacetamide and incubation in the dark for 1 h at 37°C. After incubation, 60 *μ*L of 200 mmol/L DTT (dithiothreitol) was added and allowed to incubate for 1 h at room temperature. Each sample was separated into 150 *μ*L aliquots, and aliquots were combined with 800 *μ*L of 25 mmol/L ammonium bicarbonate and 200 *μ*L of methanol. Fifty microliter of Promega sequencing grade trypsin (20 *μ*g/mL) was added to each sample and allowed to incubate overnight at room temperature. Samples were then dried using a speed vac and resuspended in a total volume of 300 *μ*L RNase-free water.

Digested samples were diluted to a final concentration of 5% acetonitrile:0.1% trifluoroacetic acid. Aliquots of 0.5% trifluoroacetic acid were added to ensure samples were acidic and pH was checked using pH strips (Sigma-Aldrich, St. Louis, MO, USA). Digested samples were desalted using UltraMicroSpin C18 silica columns (NESTGroup Southborough, MA, USA). Columns were first equilibrated by washing the column with solution A (80% acetonitrile) twice at 2000 rpm for 2 min. Columns were washed with solution B (5% acetonitrile:0.1% trifluoroacetic acid) three times. Individual samples were loaded onto each column 350 *μ*L at a time and run over the column by spinning at 2000 rpm for 2 min. Once all the sample had been loaded onto the column, the column was washed twice with solution B. The desalted sample was eluted by washing 100 *μ*L of solution A over the column, concentrated to a volume of 10 *μ*L, and resuspended in 90 *μ*L of 5% acetonitrile:0.1% formic acid. Samples were stored at −80°C until ready for analysis.

### LC-ESI-MS/MS analysis of eluates

Peptide digests were analyzed as previously described (Whitney et al. 2014) by electrospray ionization in the positive ion mode on a hybrid quadrupole-orbitrap mass spectrometer (Q Exactive™; Thermo Fisher, San Jose, CA). The Q Exactive was equipped with a nanoflow HPLC system (NanoAcquity; Waters Corporation, Milford, MA) fitted with a home-built helium-degasser. Peptides were trapped on a homemade 100 *μ*m i.d. × 20 mm long precolumn packed with 200 Å (5 *μ*m, C18AQ; Michrom BioResources Inc., Auburn, CA). Subsequent peptide separation was on an in-house constructed 75 *μ*m i.d. × 180 mm long analytical column pulled using a Sutter Instruments P-2000 CO_2_ laser puller (Sutter Instrument Company, Novato, CA) and packed with 100 Å (5 *μ*m, C18AQ: Michrom) particle. For each injection, an estimated amount of 1 *μ*g of peptide mixture was loaded onto the precolumn at 4 *μ*L/min in water/acetonitrile (95/5) with 0.1% (v/v) formic acid. Peptides were eluted using an acetonitrile gradient flowing at 250 nL/min using mobile phase consisting of: A, water, 0.1% formic acid; B, acetonitrile, 0.1% formic acid. Peptides were eluted using an acetonitrile gradient flowing at 250 nL/min using mobile phase gradient of 5–35% acetonitrile over 60 min with a total gradient time of 95 min. Ion source conditions were optimized using the tuning and calibration solution recommended by the instrument provider. Data-dependent analyses were acquired using MS survey scans in the Orbitrap followed by data-dependent selection of the 20 most abundant precursors for tandem mass spectrometry. Singly charged ions were excluded from data-dependent analysis. Data redundancy was minimized by excluding previously selected precursor ions for 60 sec following their selection for tandem mass spectrometry. Data were acquired using Xcalibur, version 2.2 (Thermo Fisher).

Tandem mass spectra were searched for sequence matches against the UniProt *P. aeruginosa* PAO1 database using Comet search engine. The following modifications were set as search parameters: peptide mass tolerance at 10 ppm, trypsin digestion cleavage after K or R (except when followed by P), one allowed missed cleavage site, carbamidomethylated cysteine (static modification), and oxidized methionine. Search results were validated by PeptideProphet probability ≥0.9 and ProteinProphet probability ≥0.95 at an error rate less than 1%.

### Hfq purification

Hfq was purified using the IMPACT Protein Purification System (NEB Ipswitch, MA, USA) using an N-terminal intein tag from plasmid pTYB21. Overnight cultures of Rosetta™ 2 (DE3) cells (NEB) carrying the pTYB21 vector with the hfq allele cloned into the multi cloning site (MCS) were diluted 1:100 into LB media containing 100 *μ*g/mL ampicillin and 12.5 *μ*g/mL of chloramphenicol and grown to mid-logarithmic phase. Hfq protein expression was then induced by addition of 1 mmol/L IPTG (Isopropyl β-D-1-thiogalactopyranoside), and cultures were grown overnight at 18°C. Cells were harvested by centrifugation, resuspended in 20 mmol/L Tris-HCl, pH 8.4, 500 mmol/L NaCl, 1 mmol/L EDTA, and lysed by sonication. Lysates were cleared by centrifugation and run through a column containing the chitin-binding domain (NEB). The Hfq protein was eluted from the column using cleavage buffer (20 mmol/L Tris-HCl, pH 8.4, 500 mmol/L NaCl, 1 mmol/L EDTA, 50 mmol/L DTT) and analyzed by sodiumdodecyl sulfate (SDS) polyacrylamide gel electrophoresis (PAGE). Contaminating proteins were removed using a 30 kDa MWCO Spin-X UF concentrator, and the wash-through, containing the Hfq protein, was concentrated using a 5 kDa MWCO Spin-X UF concentrator (Corning, NY, USA). The resulting protein preparation was analyzed by SDS-PAGE for molecular weight verification, followed by gel extraction, tryptic digest, and confirmation of Hfq identity by mass spectrometry (Table S1).

### Electrophoretic mobility shift assays

A mixture of the PrrF and PrrH RNAs was generated by in vitro transcription from PCR products of the entire prrF locus using the MegaScript Kit (Life Technologies). The native prrF1 promoter was replaced during PCR by a T7 promoter to allow transcription of the PrrF and PrrH sRNAs by the T7 RNA polymerase. Biotinylated UTP was used in a 1:3 ratio to unlabeled UTP during in vitro transcription to generate labeled PrrF and PrrH transcripts. In vitro transcription reactions were cleaned up using RNeasy Columns (Qiagen), and diluted to 4 ng/*μ*L into Hfq annealing buffer (50 mmol/L Tris-HCl, pH 7.5, 250 mmol/L NaCl, 250 mmol/L KCl) (Soper and Woodson 2008). The RNA samples were then renatured by heating at 80°C for 1 min and subsequently cooling to room temperature for 5 min. Hfq was then added to the RNA mixture at the following concentrations: 2.7, 5.4, 10.8, 21.6, 43, 86, and 172 ng/*μ*L. Binding reactions were incubated at room temperature for 20 min, then resolved by a 7.5% TGX native gel (Bio-Rad Hercules, CA, USA). RNA-protein complexes were then transferred to Bright Star Membranes (Life Technologies) using a semi-dry apparatus, and biotinylated transcripts were probed with streptavidin alkaline phosphatase (Life Technologies) and visualized by chemiluminescence using CDP-Star (Sigma-Aldrich, St. Louis, MO, USA).

## Results and Discussion

### Development of probes specific for the PrrF1, PrrF2, and PrrH sRNAs

We sought to develop a method that would identify proteins that specifically interact with either the PrrF or PrrH sRNAs in vivo. For this, we first designed cDNA probes that were specific for the PrrF1, PrrF2, or PrrH sRNAs as shown in Figure 1A. The probes for PrrF1 and PrrF2 were designed to maximize the nucleotide differences between these two sRNAs, and the PrrH probe was designed to bind to the region of the PrrH sRNA derived from the *prrF1-prrF2* IGR. Northern blots were then used to determine if these probes were specific for the PrrF1, PrrF2, and PrrH sRNAs. The PrrF1 probe only detected the PrrF sRNA in the wild-type PAO1 and Δ*prrF2* mutant, while the PrrF2 probe only detected PrrF sRNA in the wild-type PAO1 and Δ*prrF1* mutant (Fig. 1B), demonstrating that these probes are specific for their respective sRNAs. The PrrH probe detected no transcripts in the Δ*prrF2* or Δ*prrF1,2* mutants, but two smaller transcripts were detected by the PrrH probe in the Δ*prrF1* mutant (Fig. 1B), potentially due to transcriptional activity upstream of the *prrF* locus. However, as these two smaller transcripts were not detected in wild-type PAO1, the *prrF2* or *prrF1,2* mutants, we concluded the PrrH probe was specific for the PrrH sRNA.

### Identification of PrrF- and PrrH-interacting proteins

**Figure 2:**
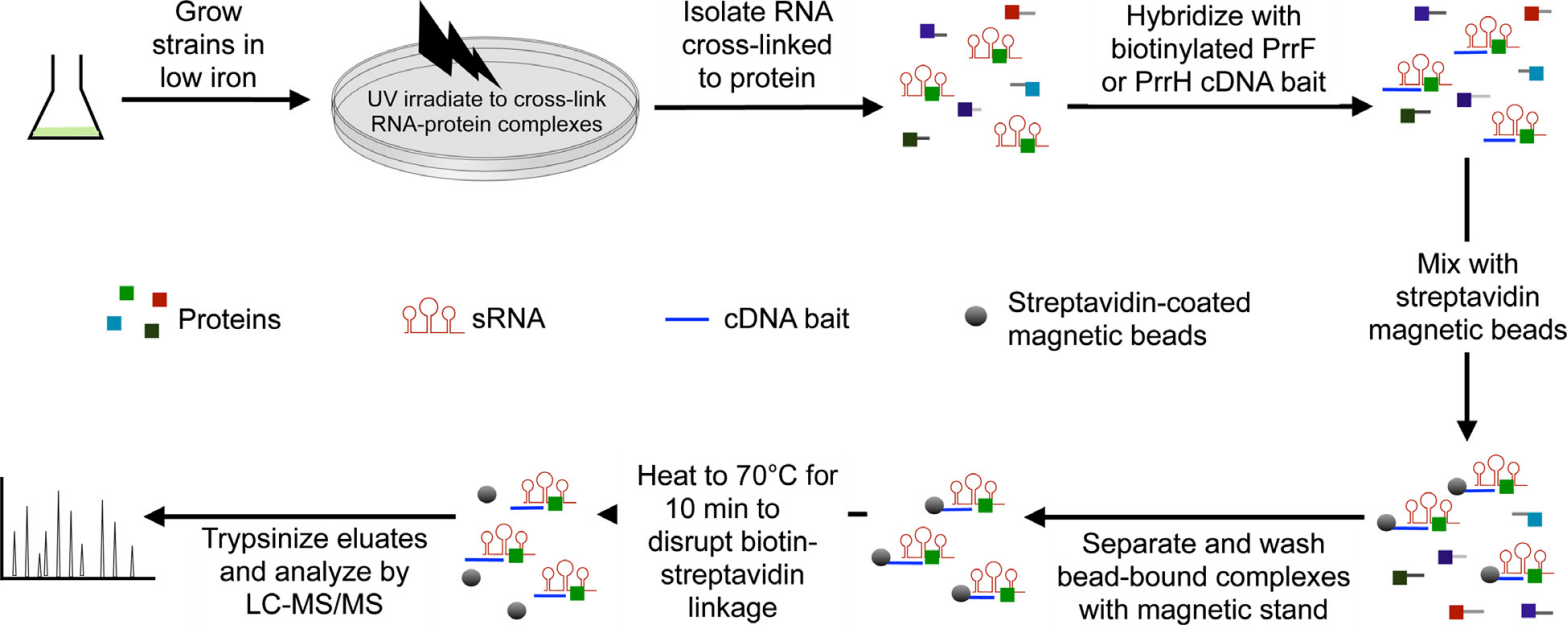
Overview of sequence-specific affinity chromatography and tandem mass spectrometry (SSAC-MS/MS) methodology. RNA-protein complexes isolated from either the PAO1 or the isogenic Δ*prrF1,2* mutant was analyzed by SSAC-MS/MS analysis, which is described thoroughly in the Materials and Methods. PAO1 samples that were enriched with the PrrF1 bait were grown in M9 medium with or without supplementation of 100 *μ*mol/L FeCl_3_. PAO1 samples that were enriched with the PrrH bait were grown in M9 medium without iron supplementation. To eliminate nonspecific interactions, the Δ*prrF1,2* mutant, grown in M9 medium without iron supplementation, was also subjected to SSAC-MS/MS analysis using either the PrrF1 or PrrH bait.

We next used UV irradiation to cross-link RNA-protein complexes in PAO1 grown in low-iron conditions. Maximal PrrF and PrrH expression was observed by qPCR at 18 h growth in M9 minimal media (Fig. 1C), so we used this time point for UV cross-linking and SSAC-MS/MS analysis, as outlined in Figure 2. Iron-depleted cultures of PAO1 and the Δ*prrF1,2* mutant were UV-irradiated to irreversibly cross-link RNA-protein complexes, and irradiated cells were harvested for RNA isolation. Purified RNA was hybridized to either biotinylated PrrF1 or PrrH cDNA probes (“bait”), which are specific for the PrrF1 and PrrH sRNAs, respectively (Fig. 1). PrrF1- and PrrH-protein complexes were then enriched using streptavidin-coated magnetic beads. Enriched protein-RNA complexes were heated to disrupt the biotin-streptavidin linkage, trypsinized, and analyzed by liquid chromatography electrospray ionization tandem mass spectrometry (LC-ESI-MS/MS). The majority of samples analyzed by this methodology resulted in greater than 15 peptides corresponding to *P. aeruginosa* proteins, with some runs producing more than 100 *P. aeruginosa* peptides. To prioritize the corresponding protein hits that were most likely to be PrrF or PrrH-binding proteins, we used the following criteria: (1) peptides corresponding to the protein had to be detected in at least three independent PAO1 samples analyzed with the same bait, and (2) peptides corresponding to the protein must not have been detected in more than one Δ*prrF1,2* mutant sample analyzed with the same bait. Samples that produced fewer than 10 peptides in total were considered failed runs and not included in further analyses. A summary of the proteins meeting our criteria for either the PrrF1 or PrrH bait are shown in Table 2, and a complete compilation of our mass spectrometry results are provided in the (Tables S2, S3).

**Table 2:** Summary of SSAC-MS/MS results

Protein	Positive samples^1^				
	PrrF1 bait			PrrH bait	
	WT − Fe (*n* = 15)	WT + Fe (*n* = 5)	Δ*prrF1,2* (*n* = 8)	WT − Fe (*n* = 8)	Δ*prrF1,2* (*n* = 9)
Hfq (PA4944)	11**	5	1	3	1
PvdL (PA2424)	5*	0	0	3	1
LysR-type transcriptional regulator (PA1128)	3	0	0	2	0
Shikimate biosynthesis (PA1750)	3	0	0	2	1
Putative oxidoreductase (PA3106)	3	0	0	2	1
HemB (PA5243)	3	0	0	0	0
HscA (PA3810)	3	1	1	1	2

Asterisks indicate a significant difference in the frequency of Hfq (*P* < 0.005) and PvdL (*P* < 0.05) positive samples when comparing PrrF1-enriched PAO1 and Δ*prrF1,2* samples, as determined by a two-tailed Student's *t*-test. SSAC-MS/MS, sequence-specific affinity chromatography and tandem mass spectrometry.

1 Number of samples with at least one peptides corresponding to the indicated protein.

#### Analysis of PrrF1-enriched samples

When we enriched cross-linked RNA samples with the PrrF1 bait, Hfq was identified in 11 of 15 PAO1 samples (73.3% hit rate), while only being identified in 1/8 of the Δ*prrF1,2* mutant samples (12.5% hit rate – Table 2). Our results with the PrrF1 bait also identified PvdL in 5/15 PAO1 samples (33.3% hit rate), and this protein was not identified in any of the Δ*prrF1,2* mutant samples (Table 2). Also identified in this enrichment were a putative LysR-type transcriptional regulator (PA1128); a protein involved in shikimate biosynthesis (PA1750); a putative oxidoreductase (PA3106); HemB (PA5243); and HscA (PA3810) (Table 2). Aside from Hfq, none of these proteins has previously been implicated in interactions with bacterial sRNAs. Statistical analysis of our results indicates that the frequency of Hfq and PvdL positive PAO1 samples was significantly higher than that of the Δ*prrF1,2* mutant (*P* values less than 0.005 and 0.05, respectively, as determined by two-tailed Student's *t* tests – Table 2). By this same analysis, none of the other proteins shown in Table 2 were identified at a significantly higher rate in PAO1 as compared to the Δ*prrF1,2* mutant.

We next performed PrrF1 enrichment on PAO1 cultures grown in the presence of 100 *μ*mol/L FeCl_3_, which should repress expression of the PrrF sRNAs. Interestingly, Hfq was identified in 100% (*n* = 5) of the PrrF1-enriched RNA samples from iron-replete PAO1 cultures (Table 2). Our interpretation of these results is that the low levels of the PrrF sRNA when PAO1 is grown in iron-replete conditions allows for interaction of nearly every PrrF sRNA with the Hfq protein, while iron-depleted conditions results in a much higher concentration of the PrrF sRNA than available Hfq protein. Strikingly, only one of the other proteins, HscA, identified in the PrrF1-enriched, iron-depleted PAO1 samples was identified in PrrF1-enriched iron-replete PAO1 samples (Table 2), and this protein was detected in only one of the iron-replete PAO1 samples. Thus, these data suggest that these proteins do not have the same affinity for the PrrF1 sRNA as the Hfq protein. Alternatively, these results could be due to decreased expression of some of these proteins under iron-replete conditions, as in the case of PvdL (Table 2). Combined with our statistical analysis, however, these data indicate that Hfq is the primary protein that interacts with the PrrF sRNAs *in vivo*.

#### Analysis of PrrH-enriched samples

We next performed SSAC-MS/MS analysis of cross-linked RNA samples enriched with the PrrH bait to determine if any distinct proteins interact with this unique sRNA. Perhaps due to lower *in vivo* levels of the PrrH sRNA as compared to the PrrF sRNA (Oglesby-Sherrouse and Vasil 2010), these experiments yielded far fewer positive hits that met our criteria than what was observed when analyzing enriched RNA-protein complexes generated with the PrrF1 bait. Nevertheless, Hfq was identified in 37.5% of the PrrH-enriched PAO1 samples, while only being identified in 11.1% of PrrH-enriched Δ*prrF1,2* mutant samples (Table 2). PvdL, PA1128, PA1750, and PA3106 were also identified in 25–37.5% of the PrrH-enriched PAO1 samples, while being identified in no more than one of the PrrH-enriched Δ*prrF1,2* mutant samples (Table 2). HscA was identified with the PrrH bait in only one PAO1 sample, and was detected in two Δ*prrF1,2* mutant samples (Table 2), indicating that detection of this protein is not likely due to a specific interaction with the PrrH sRNA. Most striking was the finding that Hfq was identified at a much lower frequency in PrrH-enriched PAO1 samples as compared to that of the PrrF1-enriched PAO1 samples (Table 2). Moreover, the rates of Hfq identification were not statistically increased in the PrrH-enriched PAO1 samples as compared to the Δ*prrF1,2* mutant. Although these results could be due to lack of statistical power in these studies, our current data are consistent with a model in which Hfq interacts more frequently with the PrrF1 sRNA *in vivo* as compared to PrrH.

### The Hfq protein interacts with the PrrF and PrrH sRNAs *in vitro*

**Figure 3:**
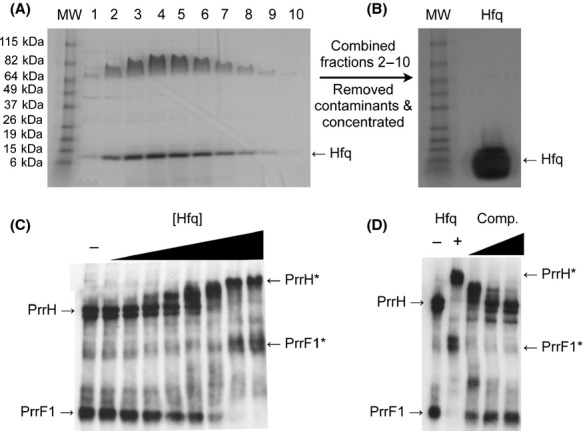
Hfq interacts with the PrrF and PrrH sRNAs. (A and B) The Hfq protein was overexpressed in *Escherichia coli* and purified as described in the Materials and Methods. Eluted fractions were analyzed by sodiumdodecyl sulfate polyacrylamide gel electrophoresis (SDS-PAGE) as shown in (A). Fractions 2–10 were then combined, contaminated proteins removed, and the Hfq protein concentrated by centrifugal filtration as described in the Materials and Methods. The resulting protein purification was verified by SDS-PAGE as shown in (B), and confirmed by mass spectrometry. (C and D) Biotinylated PrrF and PrrH RNAs were generated by *in vitro* transcription using a PCR-generated template of the *prrH* region preceded by a T7 promoter. The RNAs were diluted to 4 ng/*μ*L into Hfq annealing buffer, renatured, and combined with increasing concentrations of purified Hfq (2.7 to 172 ng/*μ*L – [C]); or with increasing concentrations of unlabeled PrrF and PrrH sRNAs (4 to 40 ng/*μ*L – “Comp.”) and a constant concentration of Hfq (172 ng/*μ*L – [D]). Binding reactions were resolved by native PAGE, and RNA-protein complexes were transferred to BrightStar membranes and detected by chemiluminescence. The PrrF1 and PrrH sRNAs are indicated with arrows. Asterisks indicate the migration of the PrrF- and PrrH-Hfq protein complexes.

To determine the validity of our SSAC-MS/MS results and further examine the interaction of Hfq with the PrrF and PrrH sRNAs, we cloned, over-expressed, and purified the *P. aeruginosa* Hfq protein as described in the Materials and Methods (Fig. 3A and B). The identity of the purified Hfq protein shown in Figure 3B was confirmed by mass spectrometry and used for EMSA's with a mixture of the PrrF and PrrH sRNAs, generated by in vitro transcription of the entire *prrF* locus. Hfq-sRNA-binding reactions were resolved by native gel electrophoresis and analyzed for mobility of the PrrF and PrrH sRNAs. These results showed a shift in mobility of the PrrF sRNAs in the presence of 44 ng/*μ*L of Hfq, corresponding to nearly a 50-fold molar ratio of Hfq to the PrrF and PrrH sRNA mixture (Fig. 3C). In contrast, shifts in PrrH mobility were detected at Hfq concentrations as low as 11 ng/*μ*L, corresponding to approximately a 12-to-1 molar ratio of Hfq and the PrrF and PrrH sRNAs, (Fig. 3C). The shifted PrrF and PrrH bands were eliminated by the addition of 20-fold excess of unlabeled PrrF and PrrH sRNA (Fig. 3D), indicating these bands correspond to a specific interaction of the PrrF and PrrH sRNAs with Hfq. Thus, our data suggest that Hfq has a somewhat higher affinity for the PrrH sRNA as compared to PrrF under these experimental conditions.

Although the EMSA's in Figure 3C are seemingly in contradiction with our SSAC-MS/MS analyses, which suggested Hfq may associate more frequently with the PrrF sRNAs versus the PrrH sRNA *in vivo*, any number of artifacts introduced during our *in vitro* analysis could be complicating these results. First, *in vitro* transcription of the *prrF* locus to generate the PrrF and PrrH sRNAs for EMSA was performed using a nonnative T7 RNA polymerase and resulted in a much higher ratio of PrrH to PrrF sRNA than what is normally seen *in vivo* (Oglesby-Sherrouse and Vasil 2010). Thus, the potential for increased interaction of the PrrH sRNA with Hfq *in vitro* as compared to *in vivo* could be due to increased concentrations of the PrrH sRNA in our EMSA's. It is also possible that altered folding of the PrrF and/or PrrH sRNAs *in vitro* as compared to *in vivo* could affect interaction of the Hfq protein with these sRNAs, particularly in consideration of the biotinylated UTP used to generate these sRNAs for our EMSA analysis. Alternatively, the low number of Hfq-positive samples obtained during the SSAC-MS/MS analysis of PrrH could be due to the fact that Hfq binding to PrrH *in vivo* precludes interaction with the PrrH bait. Finally, it is possible that additional factors, not included in our EMSA studies, modulate the *in vivo* interactions of Hfq with the PrrF and PrrH sRNAs. While these results raise several intriguing questions about how the PrrF and PrrH sRNAs interact with Hfq, they also demonstrate the capacity of the Hfq protein to bind to the PrrF and PrrH sRNAs, validating the identification of this protein by our SSAC-MS/MS analysis.

### Overall conclusions

This study developed a novel methodology for *in vivo* cross-linking coupled with sequence specific affinity chromatography and tandem mass spectrometry (SSAC-MS/MS) to identify proteins that interact with the PrrF and PrrH sRNAs. Our analyses identified Hfq, a protein known to interact with and stabilize numerous bacterial sRNAs, as a potential binding partner of the both the PrrF and PrrH sRNAs. We also identified several other proteins as potentially interacting with the PrrF and PrrH sRNAs, many of the which are involved in iron homeostasis – PvdL is required for siderophore biosynthesis, shikimate is a precursor for siderophore biosynthesis, HemB is involved in the biosynthesis of heme, and the putative oxidoreductase encoded by PA3106 likely contains an iron cofactor. The PrrF and PrrH sRNAs are not known to affect the production of any of these proteins. Thus, it is possible that enrichment of some or all of these proteins with the PrrF1 and PrrH bait is simply reflective of the colocalization of iron homeostasis factors within the bacterial cell.

However, the implications of PrrF and PrrH interacting with PvdL are particularly intriguing, as this protein is believed to be the first nonribosomal peptide synthetase (NRPS) in pyoverdine production (Mossialos et al. 2002; Visca et al. 2007). While no link has been identified between the *prrF*-encoded sRNAs and pyoverdine production, previous studies of these sRNAs have been limited to analysis of RNA levels, which would only allow identification of targets that are regulated by either transcriptional or mRNA stabilization mechanisms. It is therefore possible that PrrF or PrrH affects the production of PvdL, and perhaps other proteins, by regulating its translation, while not affecting the stability of the *pvdL* mRNA. Moreover, there exists at least one example of an sRNA that regulates gene expression by two distinct mechanisms: McaS affects mRNA stability through complementary base pairing and also regulates gene expression by direct sequestration of the CsrA protein (Jorgensen et al. 2013). Similarly, our results may indicate a role for the PrrF and PrrH sRNAs in posttranslational regulatory activities, in addition to their known function in regulating mRNA levels. Application of the SSAC-MS/MS methodology to other bacterial sRNAs of *P. aeruginosa*, as well as analysis of the interactions of these proteins with the PrrF and PrrH sRNAs, should provide additional clarity to these results.

To determine the validity of our SSAC-MS/MS results, we also purified the *P. aeruginosa* Hfq protein and analyzed its ability to interact with the PrrF and PrrH sRNAs by EMSA. These results confirmed that the Hfq protein is capable of interacting with both the PrrF and PrrH sRNAs, although our results raised additional questions about the interactions of Hfq with each of these sRNAs. More stringent biochemical and biophysical analyses are clearly required to characterize the interactions of Hfq with each of the PrrF and PrrH sRNAs, in addition to genetic analyses to determine the biological implications of these interactions. However, this study has provided critical groundwork for future characterization of Hfq interactions with the PrrF and PrrH sRNAs. Additionally, we believe that the methodology outlined in this report can provide new insights into the mechanisms by which many other bacterial sRNAs regulate gene expression, particularly for sRNAs that are not dependent upon Hfq for stability or function (Sun et al. 2002; Gaballa et al. 2008; Deng et al. 2012; Smaldone et al. 2012a, 2012b). As such, SSAC-MS/MS is a valuable tool for future studies into the mechanisms of bacterial sRNA regulation.

## Supporting Information

Table S1 MS/MS analysis of purified Hfq protein.

Table S2 Results of SSAC-MS/MS analysis with PrrF1 bait.

Table S3 Results of SSAC-MS/MS analysis with PrrH bait.
